# A Journey from Processing to Recycling of Multilayer Waste Films: A Review of Main Challenges and Prospects

**DOI:** 10.3390/polym14122319

**Published:** 2022-06-08

**Authors:** Geraldine Cabrera, Jixiang Li, Abderrahim Maazouz, Khalid Lamnawar

**Affiliations:** 1 Univ Lyon, CNRS, UMR 5223, Ingénierie des Matériaux Polymères, INSA Lyon, Université Claude Bernard Lyon 1, Université Jean Monnet, CEDEX, F-69621 Villeurbanne, France; geralcabrera09@gmail.com (G.C.); jixiang.li@insa-lyon.fr (J.L.); abderrahim.maazouz@insa-lyon.fr (A.M.); 2 Hassan II Academy of Science and Technology, Rabat 10100, Morocco

**Keywords:** circular economy, recycling, eco-design, coextrusion, multilayers

## Abstract

In a circular economy context with the dual problems of depletion of natural resources and the environmental impact of a growing volume of wastes, it is of great importance to focus on the recycling process of multilayered plastic films. This review is dedicated first to the general concepts and summary of plastic waste management in general, making emphasis on the multilayer films recycling process. Then, in the second part, the focus is dealing with multilayer films manufacturing process, including the most common materials used for agricultural applications, their processing, and the challenges of their recycling, recyclability, and reuse. Hitherto, some prospects are discussed from eco-design to mechanical or chemical recycling approaches.

## 1. Introduction

Since the discovery of polyethylene and polypropylene during the 1950s, polymers materials have been become popular and they are widely used for many applications of our daily life. Nowadays, plastic production in Europe fluctuates around 57 million tons [1]. Packaging (39.6%) and the building sector (20.4%) are the biggest end-use markets for plastics. Meanwhile, the agricultural sector represents 3.4% of the total plastic demand [1]. Between all the plastic materials applications, flexible films became very popular mainly due to their versatility, lightness, resistance, and printability. Applications of polymer films are diverse, but they are usually classified into two categories: packaging and non-packaging. The packaging products are divided into consumer (primary packaging) and non-consumer (secondary and tertiary packaging) [2]. The principal function of primary packaging is to protect the product. Then, the secondary and tertiary main purpose is to group different primary packages for easy and safe transportation [3]. Regarding the final structure of the flexible films, they can be divided into monolayer and multilayer films. Monolayer films are commonly used for tertiary and secondary packaging and less often for agricultural and building applications. Meanwhile, the structure of multilayer films is made with different layers that can be polymeric or non-polymeric materials, such as paper or aluminum foils. Using modern technologies such as coextrusion, it is possible to obtain multilayer films from 2 up to +20 layers’-layer films [4]. Nowadays, 17% of the world’s flexible film production are multilayer films, which the most popular application is food packaging [3].

Meanwhile, the non-packaging sector involves trash bags, labels, films for agriculture, construction, etc. Low-density polyethylene (LDPE) [5,6,7] and high-density polyethylene (HDPE) [8,9] are the most common polymers used in the consumer packaging sector, followed by polyethylene terephthalate (PET) [10,11,12] and polypropylene (PP) [13,14]. In the case of agricultural applications and another non-consumer packaging, LDPE and linear low-density polyethylene (LLDPE) [15,16] are the most used materials [17].

The increasing generation and accumulation of non-biodegradable waste are becoming a general popular issue since a huge amount of plastic packaging products are currently designed to have a short service life owing to the low cost and easy production [3]. Most consumers negatively perceive plastic packaging because of the considerable high amount of waste produced in their daily lives. 

According to an extrapolation from different EU countries, Plastics Europe et al. explained that in 2018 approximately 24.9% of the plastic packaging waste went to landfill and 42.6% were incinerated. Then, the remaining 32.5% of the plastic waste were recycled or exported. However, as Hestin et al. reported, the amount of waste exported out or within the EU is included in the recycled rate. Then, a recycling rate of 15% is estimated for the EU when the extra-EU exports are excluded. The recent ban on the imports of occidental plastic waste to China provides a solid argument to focus on the development of recycling inside the EU. Thanks to this, from 2016 to 2018 plastic waste exports outside the EU decreased by 39% [18].

The main objective of this review is to serve as a guide for the industrial and academic community through all the stages of plastic waste’s valorization, with a focus on the multilayer films, their design, and processing. First, the summary of plastic waste management in general is described, making emphasis on the multilayer film’s recycling process. Finally, the multilayer film’s manufacturing process and innovative coextrusion technologies are reviewed, including the most common materials used for agricultural applications, their processing, and the challenges of their recycling. As well, the concept of eco-design of multi-micro/nanolayer films is presented as a promising solution to the numerous problems that comes with the valorization of multilayer plastic film’s packaging. 

### 1.1. Polymer-Based Flexible Films Waste Management

The start-of-life phase for the polymers (virgin or recycled) used in plastic packaging applications begins with the processing via multiple converting techniques such as extrusion, coextrusion, blown extrusion, etc. Then, the first type of solid plastic waste (SPW) is generated during the manufacturing process, called post-industrial (PI) waste. This type of waste includes waste from production changeovers, fall-out products, cuttings, and trimming. In terms of recycling, the PI waste is the higher-quality grade of polymer waste, since it is clean, and the composition of the polymer is known [19]. 

Later, at the end-of-life, the product is thrown out and becomes post-consumer (PC) waste. The PC waste can be collected separately or not, depending on the country. This waste is a complex mix of polymers of unknown composition and could be potentially contaminated by organic fractions (food remains) or non-polymer fractions (paper). As expected, this PC plastic waste becomes more difficult to recycle than the PI waste [20].

Given the complexity of the challenges and the different actors that need to be involved in the recycling chain, a multitude of measures has to be implemented such as covering products design, waste collection, sorting, recycling, and end-use [21]. Once it does, the processing options for the end-use are similar for both PI and PC plastic waste. Recycling is the preferred option since it closes the loop back to the now secondary “new raw materials”. During recycling, the new raw materials can be obtained via mechanical (leading to granulates) or chemical (leading to monomer building blocks) pathways. Then, energy recovery is the greatest option in the case where the polymer waste cannot be recycled. Finally, landfill is the less-preferred option and should be avoided at all costs [20].

Considering an environmental point of view, the best alternative is to avoid the creation of SPW in general. This involves propositions of smarter packaging with eco-design or alternative materials at the production stage [22,23,24]. Naturally, all these go together with the efforts related to raising the awareness of the consumer by promoting the re-use of plastics products. However, such effort should be run in parallel to that related developing lop an efficient valorization of the huge amounts of SPW generated more and more every year [20]. Enhanced communication through the whole recycling chain, from packaging designers to the end-users, will support and help to identify possible areas of improvement, too.

### 1.2. End-of-Life Treatment of Plastic Waste

The use stage and waste treatment are always present in the lifecycle of any material. The total number of cycles that a material can submit will depend on its alteration during all stages of its lifecycle [3]. The European Union (EU) proposes through the Waste Framework Directive (2008/98/EC), the following waste management hierarchy: collection, sorting, and reprocessing [25]. Nevertheless, this waste management procedure will vary depending on the source of waste and the local implemented collection.

Over the last years, plastic waste management has been studied in many research studies [26,27,28], with rigid and mixed plastics as the main focus [29,30]. This means that the flexible films are usually considered as the non-recyclable fraction of the waste stream and in consequence sent directly to landfills or energy recovery. Most recycling companies consider that the small thickness and low bulk density of these materials can disturb the conventional recycling process. Taking into account the little information available and the not well-documented technological advances, it becomes necessary to develop cost-effective technologies for this plastic flexible waste [3].

Horodytska et al. [3] did an extensive review of the state-of-art films waste management technology, where they identified the shortcomings and established the guidelines for future research. Inspired by them, for this review, the plastic waste will be classified into post-industrial (PI) and post-consumption (PC) plastic waste, since their differences in material characteristics.

**Figure 1:**
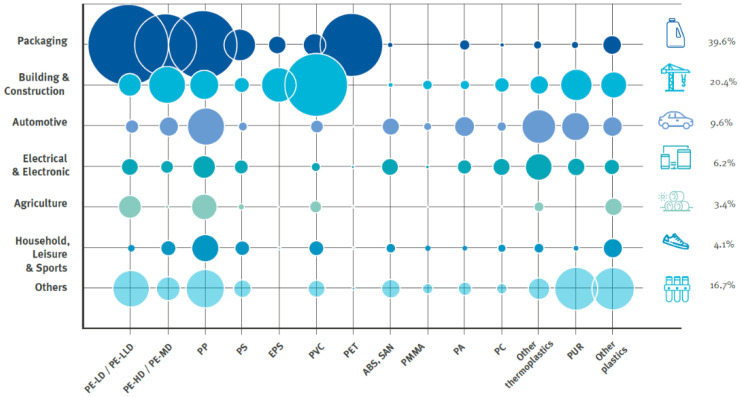
European plastics demand by segment and polymer type 2019. Reprinted from report [31]. Copyright 2020, with the permission from PlasticsEurope. (A color version of this figure can be viewed online).

In the case of PI plastic waste, the sorting stage between multilayers and monolayers is easy. However, considering the unknown origin of the PC plastic waste, it becomes more difficult to distinguish them between monolayers and multilayers. Hence, in this article, we will classify the post-consumption plastic waste as waste from the agricultural and packaging sector without the distinction of the layer’s structure. Furthermore, between the different activities involved in waste management, only collection, sorting, and treatment will be considered, since they show the most differences between rigid and flexible plastics. Then, regarding the end-of-life treatment, the plastics demand for new products gives an idea of the types of polymers that composed the bulk of the collected plastic waste. Figure 1 shows an example of the plastics demand per sector and polymer type in the EU [1]. As observed, the largest share of all PC plastic waste is packaging waste. The “big five” raw materials of high-density polyethylene (HDPE), low-density polyethylene (LDPE), linear low-density polyethylene (LLDPE), polypropylene (PP), and polyethylene terephthalate (PET) are the most common polymers used for packaging applications, which means that these polymers will dominate the composition of plastic waste. This information shows us that product design has an important impact on the recyclability (end-of-life) and the degree to which we can incorporate recycled materials (start-of-life) to new products. In design from recycling, the recycled polymer that originates the secondary raw material will be the starting point of new product development.

## 2. Circular Economy

The plastic’s circular economy is a model for a closed system that promotes the reuse of plastics products, generates value from waste, and avoids sending recoverable plastics to landfill. Plastic waste is a valuable resource that can be used to produce new polymer-based raw materials and manufacture plastic parts, or to generate energy when recycling is not viable [32]. In contrast with the linear model, the circular economy is an alternative for the industry to adopt a new plastics economy to enhance both socio-economic performances across the supply chain, while drastically reducing plastic waste. The circular economy model is based on [33]: Reduce plastic waste and pollution through product design, Retain resources and products in use, and Regenerate and preserve natural systems.

Most of the plastic materials are used for packaging and thus designed with an anticipated life expectancy of less than 1 year. Therefore, these choices in combination with the linear economy have been a major source of plastic waste, which is reflected by the accumulation of plastic waste in the ocean. Currently, a total of 150 million tons is the estimation of plastic waste found in the oceans. This proportion is projected to increase to 250 million tons by 2025, if we con-tinue to generate waste at the current rate [33].

**Figure 2:**
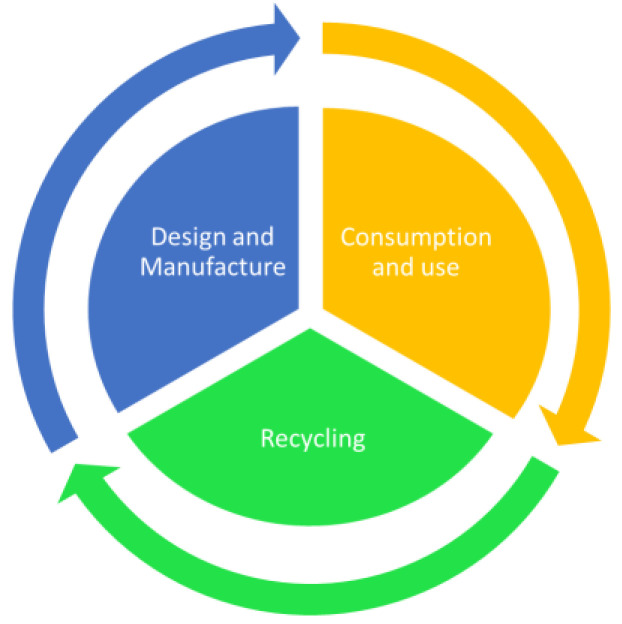
Circular economy flow diagram model.

Nowadays, the plastic industry is researching alternatives to replace fossil sources with renewable resources and carbon dioxide (CO_2_). The new strategy is all along the value chain as displayed in Figure 2: from product design to recycling, then focusing on converting more waste into recyclates, maximizing resource efficiency, and reducing greenhouse gas emissions [32,34].

## 3. Waste Collection Systems

### 3.1. Post Industrial and Agricultural Waste Collection

The post-industrial (PI) waste does not require complex collections systems since it is a clean waste of well-known composition and is normally re-used in the same company. To recycle their own scrap without leaving their facilities, many converters use developing technologies. This type of recycling allows the recovery of materials with good properties and are suitable for high-quality products manufacturing. With these types of systems, converters reduce their waste volumes and decrease the required virgin polymer amounts to be used [3].

Regarding agricultural waste, certain companies dedicated to this sector usually collect the waste on-site and then directly transport it to the recycling facilities.

### 3.2. Household Post-Consumer (PC) Waste Collection

Since the beginning, the recovery of recyclable materials has been divided into two different approaches. The first is based on source separation at individual households, and separate collection systems; the second is the recovery by mechanical processing and sorting of mixed residual waste at central establishments, which receive a larger waste flow. However, despite the efforts of the EU in the 1970s to recover valuable sources from mixed municipal waste, the poor quality of the output product proved that recycling applications were not suitable, and they risked spreading dangerous substances to the environment, such as heavy metals in compost [35].

Nowadays, to achieve the European Commission’s goal of increasing the recycling of packaging waste to 80% by 2030 (Packaging Waste Directive 94/62/EC), source separation and separate collection are the best recovery schemes to implement. In general, source separation should prevent the contamination of plastic waste by separating from other wastes at the source, and are considered as the best feasible approaches, technically and environmentally [35]. In Table 1, definitions of the different separate collections are displayed. To give an overview of the separate collection systems in plastic packaging waste, in general, the Kerbside collection (single-stream or co-mingled) and the bring/public systems runs beside the Mixed solid waste collection [35].

In the EU, the collection systems differ across the countries. Germany and Austria are considered to have the friendliest collection of flexible packaging in contrast to France and the UK. However, drop-off sites for post-consumer household films collection have become more frequent in the UK. Around 71 local authorities have kerbside collection programs for plastic flexible films. Table 2 shows a summary of different collecting schemes from the EU members. This information displayed represents the general situation in each country. Plastic film waste is generally easier to recuperate in co-mingled systems. However, the recuperation efficacy of each system will depend on the technologies implemented at the sorting facilities after the waste collection [3].

## 4. Sorting of Waste Plastic Films

In order to increase the efficiency of the recycling process, separation of collected waste is a very important step to obtain high-quality materials. Mixed waste coming from co-mingled or single-stream collection system, are generally sorted in Materials Recovery Facilities (MRF) [3].

It is important to mention that the performance of sorting and recycling varies from country to country since this step is particularly affected by the quality and output of the collection schemes and the level of contamination of the collected waste. As well, the recycling performance is related to the quality of the flows collected, especially with the pollutants found at the sorting stage in relation to the quality needed and the final end-use [21].

The technologies and equipment used by the MRFs (Materials Recovery Facilities) depend on the input waste stream received. In this article, the different sorting stages are explained depending on the collection system used to recuperate the plastic waste:

- **Mixed solid waste (MSW) collection system:** In Europe, the plants that receive mixed-waste (MSW) from householders are known as Mechanical-Biological Treatment plants, since mechanical and biological processes are used. The waste stream received consists of organic kitchen waste and recyclable materials. Different sorting stages are frequently carried out. Generally, the trommel is used for size separation and eddy current and magnets systems to remove ferrous and aluminum metals. From these plants, metals, beverage cartons, and plastics as LDPE, HDPE, and PET are the recovered materials obtained for recycling. Unfortunately, in this type of sorting, the flexible films are currently considered as contaminants and sent to landfills with the other rejects from the plants [3,35].
- **Separate collection systems:** The sorting of recyclable materials coming from separate collection programs is performed in single-stream or clean MRFs. For co-mingled recyclables, the input waste consists of plastic, glass, and paper [3]. Generally, plastic bags are removed from the waste stream at the first stage. Nowadays, the development of different mechanical equipment is necessary to facilitate the task of manual sorting by well-trained operators, which continues to be the most common method applied. At the moment, vacuum systems are installed for collecting and covering handpicked material, and bag-splitters are used to open and empty the plastics bags [36]. Then, the next stage of sorting is the separation of the flexible films (2 dimensional flat materials) from rigid heavy products (3-dimensional materials), such as rigid plastic bottles and cans. Ballistic separation is the most common well-known technology for this purpose. Air separation is an alternative technology to ballistic separation in certain applications, mostly where the feed material is relatively dry. This system relies on the fact that light material can be conveyed in an airstream at relatively low velocity, whereas heavy material will not be conveyed. However, since both ballistic and air separation technologies cannot distinguish between flexible plastic films and fiber-based material such as paper, near-infrared (NIR) optical detections are used to complete these techniques. This optical technique is based on the wavelengths reflected by the material when a NIR light hits its surface. A special sensor can determine the material type from its “fingerprint” of wavelengths [36].

In [3], the authors mentioned that results from recent studies showed that PP, PE, and mixed polyolefin can be identified by NIR. These materials can be extracted using flotation or hydroplaning, but they cannot separate from each other. Then, even if NIR is currently the most efficient technology for flexible film sorting, it has certain limitations. For example, black parts and thin coating layers cannot be detected, and neither surface between reverse printings can be distinguished [3].

Other sorting plants for the classification of recyclable materials from kerbside collection and from bringing point collection exist. For example, in Spain and Portugal, where all types of plastic and metal packaging are collected together, the sorting is performed in specific plants so-called lightweight packaging. The waste pass through different stages: reception and storage, pre-treatment, sorting of materials, and management of rejected waste. In order to remove film sheets and cardboard that can block or damage the sorting line, a pre-treatment is necessary. Then, several types of equipment such as a bag opener and ballistic separator are used, even if manual sorting is still the most common system applied. First, clean film and paper are separated by a pneumatic separator and then a magnetic one is used to remove metal items. Then, products of PET, HDPE, carton, and mixed plastics are separated by optical NIR techniques. Finally, induction separation is applied to remove the non-magnetic metals items [3].

One remaining challenge for sorting facilities is continuously changing the material composition to be sorted, which indeed requires new sorting technology. The initial product’s compositions constantly change because of the use of new mate-rials such as bioplastics, changes in the regulatory frameworks, and the constantly evolving patterns of consumption. However, as separate collection programs become more efficient, the material quality of the mixed streams drops, due to the addition of different low-quality and more problematic materials [35].

Recently, Schmidt et al. [37] studied the quantity and composition of multilayer packaging contained in the post-consumer waste stream. They showed that multilayer packaging is not assigned to any specific sorting fraction, since there is a lack of large-scale industrial sorting and recycling process. In consequence, multilayer packaging is dispersed into various recycling paths such as films, mixed plastics, or residual material. Therefore, due to a multitude of different packaging solutions on the market and the sometimes too-small quantities of some multilayer packaging types, there are no economic processes for its recycling.

## 5. Plastic Films Waste Treatment

After the collection and sorting process, different recycling processes can be applied for the flexible plastic films waste treatment process. Mechanical recycling [19,38,39], chemical recycling [40,41,42], and energy recovery [43,44] will be discussed in this review.

**Figure 3:**
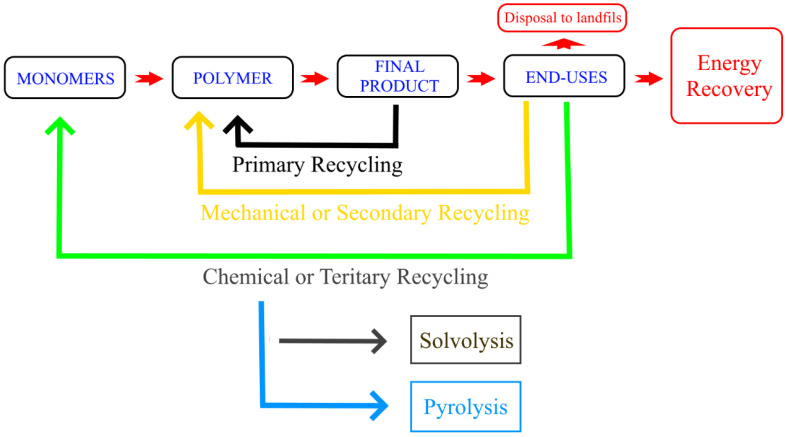
Schematic representation of the recycling methods.

Methods of recycling are generally divided into four categories: primary, secondary, tertiary, and quaternary (Figure 3). Primary recycling is considered when the materials after recycling present equal or improved properties compared to the initial or virgin materials. On the other hand, when the recycled material obtained presents worsened properties than the virgin material, the method is called secondary recycling or down-cycling method. In the tertiary (also known as chemical or feedstock) recycling method, the waste stream is converted into monomers or chemicals that could be advantageously used in the chemical industries. Finally, the quaternary (also known as thermal recycling, energy recovery, and energy from waste) recycling method correspond to the recovery of plastic as energy and is not considered as recycling in a true circular economy. It is important to note that there is a hierarchy in these four recycling methods, where the mechanical recycling is to be implemented first. Plastics Europe et al. [45] believe that adopting this hierarchy of recycling technologies, from mechanical to chemical recycling, offers the value chain optimal circularity, coupled with lower environmental impact.

In the context of circular economy thinking, the recycling of materials can be also categorized based on the product, which is manufactured from the secondary raw materials:

- **Closed-loop recycling:** Recycled materials are used to produce the same product from where they were originally recovered. Only recycled plastics or a blend between recycled and virgin plastics can be used to produce a new product. This type of recycling ensures that the product can be recycled continuously, and its recovered material can be added at the same rate [20].
- **Open-loop recycling:** Recycled materials are used for different applications than the product they were originally recovered from. However, this does not imply that the new application is of “lower value” [20].

These two terms will be used in this article to classify the recovery materials obtained from different plastic packaging waste (post-industrial, post-consumption agricultural, and packaging).

### 5.1. Mechanical Recycling

**Figure 4:**
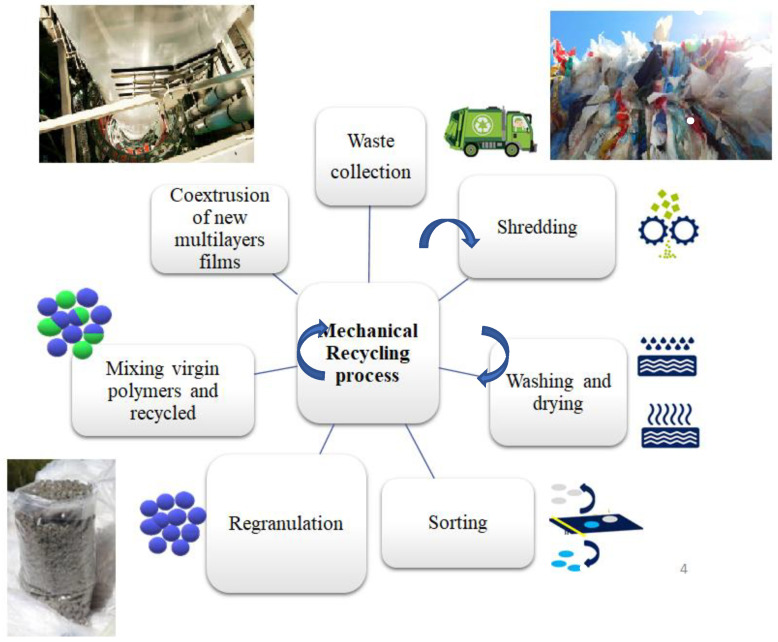
Schema: Example of a multilayer film’s mechanical recycling process. Pictures of plastic waste obtained from the website of “Barbier Group” [46]. This figure was generously shared by Mr. Gerard Pichon from “Barbier Group”.

Mechanical recycling (Figure 4) is the most common recycling technique applied for solid plastic waste (PI and PC), and it is carried out by different mechanical processes where the polymer structure remains unchanged. In terms of quality, the recycled material from closed-loop processes is very close to the original material. Consequently, these materials can be used as secondary raw materials for high added-value products manufacturing. The input waste normally consists of products from a single type of plastic and vaguely contaminated. The recycling process is composed of waste transformation by extrusion, where the plastic is melted and re-granulated. Decontamination methods can be also applied before the re-granulation [3].

In the case of the open-loop mechanical recycling process, the input waste is a single type of polymer material or a blend of compatible polymers. As shown in Figure 4, mechanical recycling consists of different processes such as:

- Separation and sorting: based on shape, density, color, or chemical composition.
- Baling: for ease handling, storage, and transport, the plastic waste is fed into a baler where it is compressed into bales.
- Washing and drying: elimination of contaminants.
- Shredding: size reduction of the product to flakes.
- Compounding and pelletizing: reprocessing of the flakes into granulates.

The previous steps can be applied anywhere between, multiple times, or not at all, depending on the type of plastic waste. For example, sorting is applied more often to post-consumer waste (PC) than to post-industrial waste (PI), since the latter tends to be greater separated in advance in terms of its composition. Then, since the post-consumption (PC) waste stream contains contaminants such as plastic additives, inks, and remnants of incompatible polymers that affect the plastic properties during reprocessing, they are suitable only for less demanding applications: trash bags, pipelines, products for agricultural applications, etc. [3].

### 5.2. Chemical Recycling

In this recycling methods, plastic waste is considered as raw material for the production of valuable products such as monomers or petrochemical feedstocks. Various processes can be considered for chemical recycling, and they present different levels of maturity. Nowadays, plastic waste is considered a promising raw material for the production of fuels and chemicals. The interest is the production of valuable products such as monomers or petrochemical feedstock. The most known processes are gasification (partial oxidation), pyrolysis, hydrogen technologies, fluid-catalytic cracking, and depolymerization (methanolysis, glycolysis, and hydrolysis). Both types of chemical recycling consist of monomer recycling or feedstock recycling and are considered as ideal methods for the protection of the environment by the reduction of the non-degradable waste volume [20]. For polyolefins, pyrolysis is the most common technique used. The products obtained from pyrolysis are liquid and gas that enclose the substances of interest. However, since the separation costs are very high, the recycled products are used as fuel. A summary of the most used chemical recycling techniques is displayed in Table 3, including their challenges and advantages.

It is important to mention that most of the chemical recycling techniques of plastic waste are still at early stages and they are not expected to be completely operational before 2025 [21]. However, they have high potential for the valorization of contaminated and heterogeneous plastic waste, where the separation and sorting are not viable economically and technically. Therefore, as reported by Plastic Europe et al., a 7.2 billion euro’s investment is expected from great polymer producers by 2030. Subject to European regulatory constraints in favor of waste reduction and the circular economy, producers aim to move from a production of 1.2 million tons of recycled plastics in 2025 to 3.4 million tons in 2030 [45].

### 5.3. Energy Recovery

Energy recovery consists of the burning of waste to produce energy in the form of electricity, heat, and steam. Due to their high calorific value, plastic products are considered as a promising source of energy, since they are derived from crude oil. The production of water and carbon-dioxide after combustion make them similar to other petroleum based-fuels [47]. The volume of waste can be reduced by 90% after the incineration, which is an advantage when the landfilling is limited, and the lack of space becomes important. There are different incineration methods used for the plastic waste [3]:

- One stage: co-incineration of municipal solid waste with high fractions of plastic waste
- Two-stage: fluidized bed combustion process
- Cement industry: plastic solid waste commonly used as a fuel in cement kilns in order to save energy and reduce costs

Even with notable differences between the member states of the EU, there is a general trend of redirecting plastic waste from landfilling to incineration due to the now-strict measures concerning landfilling imposed by the EU legislation. In consequence, most of the low-quality waste is channeled to energy recovery facilities, whose capacity is in constant increase [21]. However, an important number of environmental concerns are associated with the incineration of plastic waste. The combustion of synthetic polymers such as PET and PE can generate volatile organic compounds, smoke, particulate-bound heavy metals, polycyclic aromatic hydrocarbons, and dioxins, which have been identified in airborne particles from the incineration [47]. Moreover, despite the economic benefits, energy recovery is not in resonance with the circular economy principles. Therefore, the recycling and reuse process are prior for the plastic waste management. Energy recovery should be applied only to the non-recyclable fraction [3].

### 5.4. Post-Industrial Plastic Film Waste Recycling

In the EU, the recycling rates of the post-industrial (PI) plastic waste are higher compared with the household or post-consumer (PC) waste. The large volumes of industrial waste and the known composition create most cost-effective recycling and facilitate the production of purer recyclable materials [21]. In this context, many companies have been investing into different recycling tech-nologies in order to recycle their own production scrap. This type of recycling system leads to recovery materials with good properties and appropriate for high quality products processing. Then, since their composition is known, the sorting stage becomes easier between monolayers and multilayers systems.

In the last two decades, the formulation and production of biodegradable and/or bio-based polymers, as an alternative to the synthetic counterparts, is a viable strategy towards sustainability in a green future. Despite the interest in the growth of biopolymers, their waste stream is limited. Most of them are polylactic acid (PLA) and PLA-based composites which are usually not collected separately [48]. Biodegradable polymers are susceptible to be broken down into simple compounds because of microbial action, and different bioplastics have been known to undergo this process in a reasonably short time (e.g., six months), and are commonly identified as biodegradable. In some specific cases, mechanical recycling might be chosen as a priority compared with the biodegradability process, since it has been demonstrated that the mechanical recycling process is more environmentally friendly. Cosate de Andrade et al. [49] presented a Life Cycle Analysis of PLA comparing chemical recycling, mechanical recycling, and composting, and they found that mechanical recycling had the least environmental impact, followed by chemical recycling and, lastly, composting, when considering the climate change, human toxicity, and fossil depletion categories. Whereas the recycling of bio-based polymers and their composites must be further investigated and better addressed, considering the target applications for these materials and for a successful recycling process, the bioplastic and their composites waste stream must be collected separately to the other plastic waste stream [23,34]. Furthermore, we have to consider also that those bio-based materials are very sensitive to humidity, thermal, and/or hydrolytic degradation, and the loss of their physical, mechanical properties is noted up to the various steps of mechanical recycling. As reported in the recent review of Morici E. et al. [34], only homogeneous (bio)polymers-based materials could successfully perform the chemolysis through glycolysis, aminolysis, methanolysis, alcoholysis, and hydrolysis. For heterogeneous biobased materials, the cracking and gasification could be considered more appropriate methodologies. The collected (bio)plastic materials, having an extremely heterogeneous nature, could be successfully recycled through energy recovery. 

#### 5.4.1. Monolayer Film’s Recycling

The post-industrial (PI) waste coming from monolayer films is recovered by the mechanical recycling process. Depending on their characteristics, they can be recycled by closed or opened-loop. The non-printed monolayer film’s scraps are recovered by closed-loop recycling since they are clean and homogenous. In the case of printed scrap, only a closed-loop recycling is possible in order to avoid important quantities of waste going to landfilling.

Nowadays, many advanced technologies are developed to obtain recycled materials coming from surface printed plastic waste. Some of these technologies involve filtration, homogenization, and degassing stages, including final extrusion of recycled pellets. *EREMA*, which is a leading plastic recycling company, has patented the latter mentioned technologies. However, because of the poor properties of the final product obtained, the recycled material is only adequate for less demanding applications such as trash bags, plastic lumber, etc.

Horodytska et al. did extensive research concerning the different deinking methods available and their advantages and disadvantages. Focusing on water-based inks, many researchers have investigated how to eliminate the ink from polyethylene films by using different surfactants [3]. At the beginning [50], concluded that cationic surfactants were the most effective (over a pH range of 5–12) at deinking. On the contrary, anionic surfactants had almost no deinking effect even at high pH levels. Then, in the case of nonionic surfactants, the deinking process is possible depending on the pH level of the solution. Eventually [51] investigated the deinking of solvent-based ink from polyethylene films. They found that cationic surfactants were the most effective, which is the same case as water-based inks. The only difference is that a minimum pH level of 11 is required for water-based inks [51].

**Figure 5:**
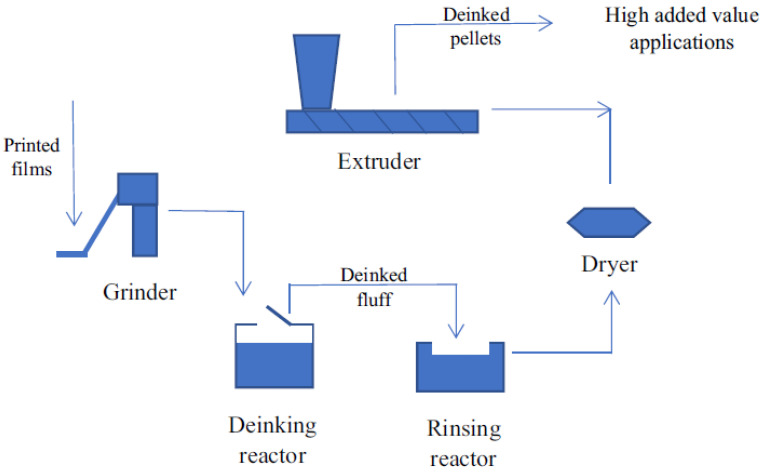
Cadel Deinking process of printed films, developed by the University of Alicante. Reprinted from publication [2]. Copyright 2018, with the permission from Elsevier. (A color version of this figure can be viewed online).

Later, researchers from the University of Alicante developed an innovative deinking process to remove the ink from plastic’s surfaces [50]. Since they proved that this process was economic and technically viable, they settled a semi-industrial deinking plant called Cadel Deinking. Figure 5 describes the deinking process with a water-based solution where no environmentally dangerous chemicals are used. In order to obtain recycled plastic free of ink with a good quality, the printed film goes through different steps such as grinding, deinking, washing, drying, and pelletizing. Finally, the recycled pellets obtained can be used for high added value product processing. Moreover, they designed a water treatment system in order to reduce the consumption and to recover the deinking chemicals [3].

**Figure 6:**
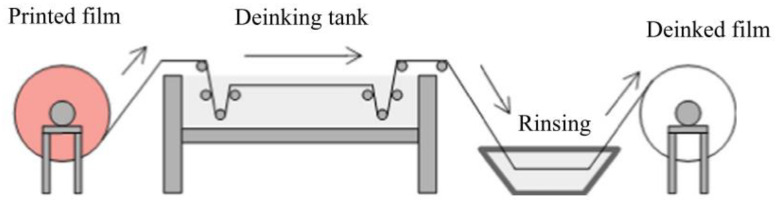
Gamma Meccanica deinking process. Reprinted from publication [3]. Copyright 2018, with the permission from Elsevier. (A color version of this figure can be viewed online).

Gamma Meccanica is an Italian company who also developed a deinking technology for plastic flexible films. The process is limited to plastic film rolls, as can be observed in Figure 6, which is not ideal since most of the printed waste films have defective products such as shopping bags, packages, etc. As a result, a grinding stage is necessary to cover a large number of film waste. In Table 4, a summary of the different deinking processes available in the world are described and explained. The *CLIPP+* is a European-funded project where carbon dioxide in supercritical conditions is used for deinking and deodorization of PI polyolefins films. The latest results showed that the recycled films obtained could be used in secondary packaging applications [3].

Chemical recycling of post-industrial (PI) monolayer film scrap had also been studied, as pyrolysis in LDPE plastics bags. Liquid fractions with hydrocarbons could be obtained in the domain of commercial gasoline. However even if the technique is environmentally friendly, large amounts of waste are required to reduce the functioning costs [3].

#### 5.4.2. Recycling of Multilayer Films

Multilayer films are made of different types of polymers for synergic properties as well transparency, gas and water barrier properties, stiffness, flexibility, etc. For the clarity purpose, a specific section of this chapter is dedicated to their processing and a wide range of applications. By consequence, the recycling of these films becomes more challenging compared with monolayer films. Through the past years, different recycling methods such as compatibilization, delamination, and dissolution-precipitation have been investigated [2,3,52,53]. Since complex blends are obtained after the recycling process, compatibilizers are added into the blends in order to increase the cohesion between the different polymers. For example, DuPont has developed a variety of compatibilizing resins such as Fusabond^®^ for film applications [54]. In addition, Dow Chemical Company has also developed polymer modifiers such as RETAIN^®^, in order to facilitate the recycling process of barrier films that contain EVOH or PA [55].

The mechanism of multilayer delamination consists of the dissolution of macromolecules. Physically, chemically, or mechanically, the delamination can be induced by the decomposition of the interlayer or by reactions at the interface [2]. The segregation of the different layers of the films and the recycling of polymer blends are done separately. The polymers used as “tie-layers” to join two layers (usually made with incompatible polymers) are usually removed with a determined solvent. For example [56] investigated the recycling of multilayer films containing PE, aluminum, and PET. In order to delaminate the multilayer film, Acetone was used, and PET was depolymerized with ethanol in supercritical conditions. Then, since the solvents were recuperated by distillation, the process was considered not harmful for the environment [56].

In the case of inked multilayered film, researchers also worked on a combined recycling process of delamination and deinking. They determined that delamination should be applied first, since sometimes the ink deposits between the layers (mostly with food contact applications). However, in order to reach higher recycling rates, a correct sorting of delaminated polymers before the extrusion is necessary [3].

The selective dissolution-precipitation technique is a mechanical recycling method used to separate and recycle the polymers using solvent or non-solvent systems. A separation step is necessary after the dissolution of the polymers, using the differences in material densities [3].

**Figure 7:**
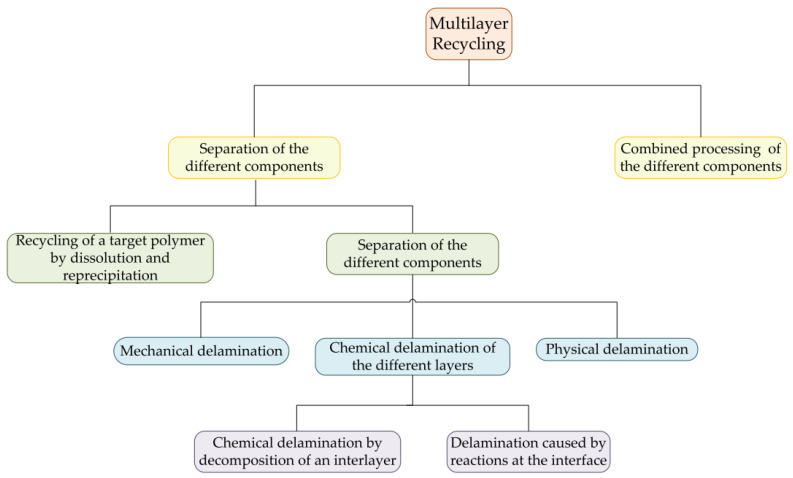
Summary of the introduced recycling methods of multilayers films waste. Adapted from [2]. (Copyright 2022, Elsevier).

Figure 7 displays the summary of the current advances in research. It describes two paths to recycling multilayered films. The first path is the separation of the different multilayer polymers in separated recycling streams, in order to make them suitable for recycling. The second path is the processing of the used polymers together in one compatibilization stage [2]. As explained before, compatibilization steps consist of the addition of suitable molecules that work as compatibilizers. Since post-industrial waste has a known composition, they are suitable to be recycled by compatibilization.

The separation methods of the different polymers presented on the multilayer are sub-divided in two strategies: (i) Separation of selective polymers by the dissolution-precipitation method and (ii) physical, mechanical, or chemical delamination method. 

In general, physical delamination methods consist of the dissolution of the interlayer (tie-layer) using solvents, water, or aqueous solutions with a specific pH value. The mechanical delamination is a less used method since usually a strong adhesion between the layers is present. In the case of chemical separation methods, there are two types: delamination by the decomposition of an interlayer and by the induction of reaction at the interphase [2].

More recently, Ref. [57] presented a new approach from eco-designed to recycling of the multilayer structures as an alternative way to improve the valorization process, assuming that their mechanical recycling is possible. This recent work describes a future-oriented approach for the recycling of polyethylene-based multilayer films. The method involves going from eco-design to mechanical recycling of multilayer films via forced assembly coextrusion. This study’s originality consisted in limiting the number of constituents, reducing/controlling the layer’s thickness, and avoiding the use of tie layers as compatibilizers. The ultimate goal was to improve the manufacturing of new products using recycled materials by simplifying their recyclability [57]. Based on the results obtained, a proof of concept was demonstrated with the eco-design approach of multi-micro/nanolayer films as a very promising solution for the industrial issues that arise with the valorization of recycled materials.

### 5.5. Post-Consumer Plastic Film Waste Recycling

As explained before, considering the diverse and unknown composition of the post-consumer plastic waste, it is difficult to separate them into monolayers and multilayers. Thus, in this article, they will be classified as waste from agricultural and packaging sectors.

#### 5.5.1. Agricultural Plastic Waste (APW)

Plastic products for the agricultural sector represent almost 4% of the total plastic product consumed in Europe and the USA. In the North European countries, agricultural films are mainly used for silage and bale wrapping films; meanwhile, in the South countries, green houses, low and high tunnel and mulching are predominant [58]. However, an increased accumulation of plastic waste in rural areas is increasing thanks to the expanding and extensive use of plastics in agriculture. In the past years, most of this waste was sent to landfill or burnt uncontrollably by the farmers, which released harmful substances to the environment, and affected the human health and the safety of the farming products [59].

The majority of the plastics films for agriculture are made mostly of low-density polyethylene (LDPE), due to its relatively good mechanical and optical properties. Then, high-density polyethylene (HDPE) and ethylene-vinyl acetate (EVA) are common polymers also used for some agricultural applications. As mentioned before, since the plastic production for agriculture applications represents a minor percentage of the total plastic production, this fact facilitates the collection of the plastic waste. The agricultural polymer-based films used in a specific rural region are similar since the same cultivations take place. Then, the APW (agricultural plastic waste) generated at a regional level is homogeneous, since it is concentrated geographically and generated at specific periods each year, with the exception of bale wrapping films, silage films, and other related plastics. Nevertheless, even if the APW waste management is easier than other post-consumer plastic waste, the APW is heavily contaminated with pesticides, soil, stones, vegetation, and other organic waste. Of course, the contamination level depends on the applications, management during their use, removal practices, and storage conditions of the plastic waste in fields [59].

**Figure 8:**
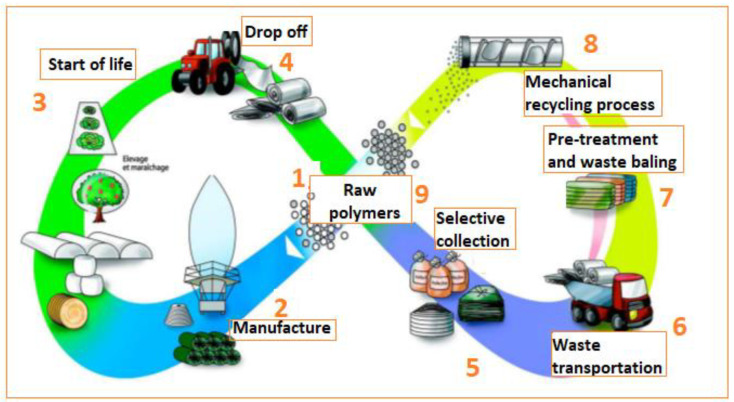
Schematic of the agricultural plastics film’s life cycle in the context of a circular economy. Adapted from [60].

The most justified recycling method for the APW is mechanical recycling from a financial and environmental point of view. In the case of the non-recyclable waste, energy recovery in cement kilns is the most common practice used. In Figure 8, an example of the agricultural plastic film’s life cycle in France is displayed, in the context of a circular economy. The disadvantage of the mechanical recycling process is the contamination with dirt, soils, etc., which obliges the recycler to include a washing step and sometimes even a pre-washing before the waste starts to be processed. This washing step increases the costs and the need of water and energy. However, it is possible to reduce the contamination during the baling of the plastic waste. On the other hand, even if the energy recovery process is an efficient alternative to the APW management, it is a controversial subject of public concern due to the potential contributions of gases combustion to the atmospheric pollution. Currently, regulation and technical specifications are being developed among the European countries [58].

Mechanical recycling and the APW quality.

The post-consumer APW mechanical recycling is preferred due to the homogeneity of the films and single polymer waste available. The films go through different steps during the mechanical recycling: washing, shredding, drying, and pelletizing [3]. In order to summarize and evaluate the recycling practices of APW, it is important to understand the factors that limit their recyclability:

- **Inert contaminants:** The contamination by sand and soil of the PE-based APW is the reason why this waste is not commonly recycled with other PE plastic wastes. Besides the high cost of the washing stage, the water is usually trapped in the folds and is not easily removed during drying. Prolongation of the drying cycle degrades the plastic and consumes energy. Additionally, stones and soil can damage the blades for the cutting and processing equipment that increases the maintenance costs [58].
- **Thickness:** Most of the recycling industries in Europe indicated that the thickness of the APW limits the productivity of the mechanical recycling lines. It was shown that the productivity of the recycling process decreases from 1000 kg/h for a 40 µm average thickness film to 500 kg/h for a 20 µm film [58].
- **Co-mingled plastics:** A poor sorting induces the contamination of the PE-based APW with other polymers; for example, PE based films mixed with PP agrochemical bottles. The formulation of the plastic films also affects the sorting. In the case of the multilayer films made with PE and EVA, there is no problem since PE and EVA are compatible and even the latter helps the recycling process by increasing the Melt Flow Index (MFI). However, PE is not compatible with PP, polyamide (PA), polycarbonate (PC), etc., which have different melting points than PE. This reasoning highlights the importance of preventing cross-contamination between the sorted piles throughout storage in the field, transportation, and baling [59].
- **Ageing (ultraviolet radiation):** Photodegradation of the plastics is caused by the exposure to ultraviolet light (UV), which affect the recyclability. Chain scission and crosslinking phenomena affect mostly the rheology of the molten mixture [61]. The chain scission reduces the molecular weight; meanwhile, the crosslinking increases the bonding between polymers chains, which leads to a molecular weight increase. The formation of carbonyl groups and vinyl groups are the major functional groups that accumulate with the photodegradation of PE [61].

Briassoulis et al. [58] did an extensive investigation about the quality of agricul-tural plastic waste’s characteristics after their mechanical recycling. They found that the inadequate sorting of the plastic waste (per thickness, color, and category) is an important factor that affects the quality and commercial value of the APW. They also demonstrated that the exposure of the APW to the sun radiation under normal field conditions (for the typical time periods) is not intense enough to lead the plastic to a severe degradation that make the APW non-recyclable, with an exception in the case of the mulching films. In cases where mechanical recycling is too expensive because of the sorting high costs, drying stages, or the low quality of the waste material, energy recovery is the commonly selected option to avoid landfilling [3].

Then, La Mantia [62] investigated the possibility to use the recycled material obtained from greenhouse covering in closed-loop. They obtained the best results with virgin and recycled material monolayer blends and with coextruded blends where the recycled material was placed between two virgin layers.

Most recently, Cabrera G. et al. [63] studied the valorization of post-consumer agricultural waste films, specifically bale wrapping multilayer films. These waste films go through different processes that involved the following: waste collection, recycling, and reuse of the multilayer waste films as a secondary raw material for new applications. However, after the recycling process, they observed a migration phenomenon of certain additives, which represents a drawback for the industrial process and limits the reuse of the waste films as a raw material. After an exhaustive study, they proved that the use of mineral fillers is an excellent solution to avoid the migration of these additives and therefore increase the recyclability of bale wrapping multilayer films. 

#### 5.5.2. Packaging Plastic Waste 

According to Horodytska et al. 2015, in Europe approximately 100 billion plastic bags are consumed every year, meanwhile, only 7% are recycled [3]. However, enormous efforts have been made to reduce the consumption of retail bags and other wrap films. High quality materials can be obtained from the recycling of retail bags and other wrap films. If the waste is clean and uncontaminated, it can be suitable for the same applications as the original [3].

Unfortunately, flexible film waste coming from Kerbside collection is commonly considered for the recyclers as a contaminant and in consequence is removed from the waste stream. Even if sometimes it is used for energy recovery, most of the time it is sent for landfilling. For example, the household waste includes primary packaging that are usually multilayer films. Nevertheless, since it is not possible to separate monolayer from multilayer, all the flexible films are rejected from the waste stream and considered as a contaminant [3].

The majority of the plastic films are made from high-density polyethylene (HDPE), low-density polyethylene (LDPE), and linear low-density polyethylene (LLDPE). The authors of [64] recovered the data of post-consumer plastic film collected in the United States in 2017. For the 2017 survey, they used the following film categories:

- **PE Clear film:** Clean PE film from commercial sources, including stretch wrap and polybags.
- **PE colored film:** Mixed color PE film from commercial sources, stretch wrap but no post-consumer bags.
- **PE retail bag and film:** Mixed color, clean PE film, stretch wrap and retail collected post-consumer bags, sacks, and wraps.
- **Kerbside collection films:** Post-consumer PE mixed film collected in kerbside and sorted at materials recovery facilities (MRF).
- **Other PE films:** PE film that does not fit in any of the previous categories.
- **Other non-PE films:** Non-PE film that include polypropylene (PP) and polyvinylchloride (PVC).

MOORE Recycling Associates [64] reported that in 2017 the number of plastics bags and film recovered for recycling was 1 billion pounds, which represents an increase of 54% since 2005. They explained that depending on the collection system, recovered film bales can contain a combination of HDPE, LDPE, and LLDPE resins or can contain a single resin. For example, stretch films or pallet wrap can be collected separately and sorted as PE clear film or it may be mixed with other polyethylene films. Then, plastic bags and wrap are commonly mixed with stretch films and other retailer-generated scrap film, in order to obtain an efficient collection at retail collections. Hence, “bags only” bales containing only bags and wrap are not common. Thus, the total amount of recovered post-consumer bags and packaging were defined as the combined total of Kerbside collection films with a specific percentage of the PE retail bags and films bale.

**Figure 9:**
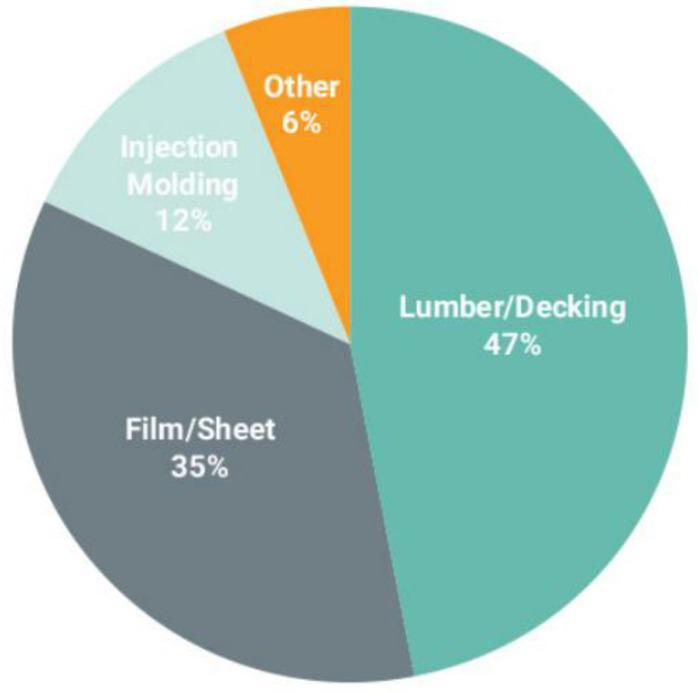
Reclaimed end-uses for U.S post-consumer plastic packaging in 2017. Adapted from [64].

The majority of the US film processing plants are for clean PE films, which can be easily used as raw material for a new product without the washing step, or for a single polymer film (only LDPE). Films, sheet, and composite lumber production are the main uses for recycled post-consumer films, as shown in Figure 9. As observed, composite lumber remains the preferred domestic end use market for the post-consumer films [64].

Achilias et al. [65] reported that the dissolution/precipitation technique is an effective process in recycling waste packaging material. The proper experimental conditions as type of solvent/non solvent, polymer concentration, and dissolution temperature were selected based on the model polymers (HDPE, LDPE, PP, PS, etc.). They obtained good polymer recovery in most of the waste samples studied. However, lower recovery values obtained in other samples were attributed to the removal of additives that were present in the original structure [65].

To sum-up, there are two fundamental needs to improve the packaging film’s recycling:

- **Higher demand to absorb the material currently collected:** Recyclers are struggling to compete with virgin polymer suppliers. Without the appropriate demand, the improving of the collection and processing in fractures becomes more expensive and difficult [64].
- **More education on recycling:** With the lack of general awareness, the quality of the drop-off film recycling streams and Kerbside recycling struggles. It has been demonstrated that consumers do increase participation if they are conscious that they need to recycle [64].

Concerning chemical recycling, a company named RES Polyflow™ patented a process to obtain petroleum products from mixed plastic waste. As reported by them, the blend between rigid and flexible plastics can feed a reactor. Molten fluids, condensable petroleum gas, and other gases can be produced through the reactor, under certain heat and anaerobic conditions [3].

Nowadays, plastic film producers are committed to a sustainable environmental development. Many efforts have been made to increase the recyclability of the materials. In consequence, the multilayer film design (for different application) is done considering the end of life of the product [35].

## 6. Processing of Multilayer Films and Applications

This section provides an overview on the research work that has been carried out in the academia in the past years regarding multilayered polymers from coextrusion process. The state-of-the-art methods for fabricating multilayered polymers are firstly introduced and compared. A specific attention is given to their applications in regard to the agriculture sector as well their recyclability. 

### 6.1. Coextrusion: Principle and Technologies

Coextrusion is an industrial process widely used to form multilayered sheets or films that are suitable for various products, ranging from food packaging, the medical area, and recently in microelectronic and nonlinear optics with more than thousands of layers [66,67]. Multilayered polymer systems are produced to satisfy specific requirements for high value-added applications such as gas barrier films [68,69], mechanical robust systems [70,71,72,73], and optical applications [74,75,76]. The coextrusion is a process which combines multiple polymers via two or three extruders using a feedblock system, where the polymers melted (from separate extruders) are brought together [77]. Each component of the multilayer structure provides its own end-use characteristics.

A typical example is oxygen-barrier food packaging. This kind of packaging material has usually a polyethylene (PE) or polypropylene (PP) based structure with an oxygen-impermeable polymer such as ethylene-vinyl-alcohol (EVOH) or polyamide (PA) as a central barrier layer. Since the barrier polymers have normally poor adhesion to the main structure polymers, copolymers are used as tie-layers in order to compatibilize and improve adhesion between the barrier and the external layers [78].

Over the last five decades, continued research and development in the academic and industry domains allowed a continual growth and expansion of the micro and nanolayered film coextrusion technology [79] to commercial relevancy. Meanwhile, many interfacial and rheological phenomenon during the co-extrusion and forced assembly are also deeply studied [80,81,82,83]. Nearly 500 issued patents for composition of micro or nanolayered materials applications have been published between 2000 and 2010 [84]. In order to increase the research and commercialization of the advanced microlayer technology, research in polymer processing have been developed as well as the advancement of coextrusion feedblocks and layer multiplying die manufacturing.

#### 6.1.1. Cast Film Coextrusion

A combination of two or more extruders through a multichannel-layered feedblock is used to produce conventional 2 to 17 layered cast films. Polymer materials separated by different streams are combined into parallel layers in the feedblock before exiting to a film, sheet, or annular die. Polymer dies companies such as Cloeren, Nordson, Macro Engineering, etc. produce multilayered polymer feedblocks which are up to 32 layers. Over time, multilayered polymer films with less than 20 layers have been produced in order to tackle complex blends film’s extrusion process, due to the performance and cost factors such as those listed by [84]:

- Potential reduction in expensive polymer materials by controlling the polymer domain location, continuity, and thickness.
- Incorporation of recycled materials at the internal layers without degrading the film properties.
- Reduce the film thickness maintaining the mechanical, transport, and/or optical film properties.

**Figure 10:**
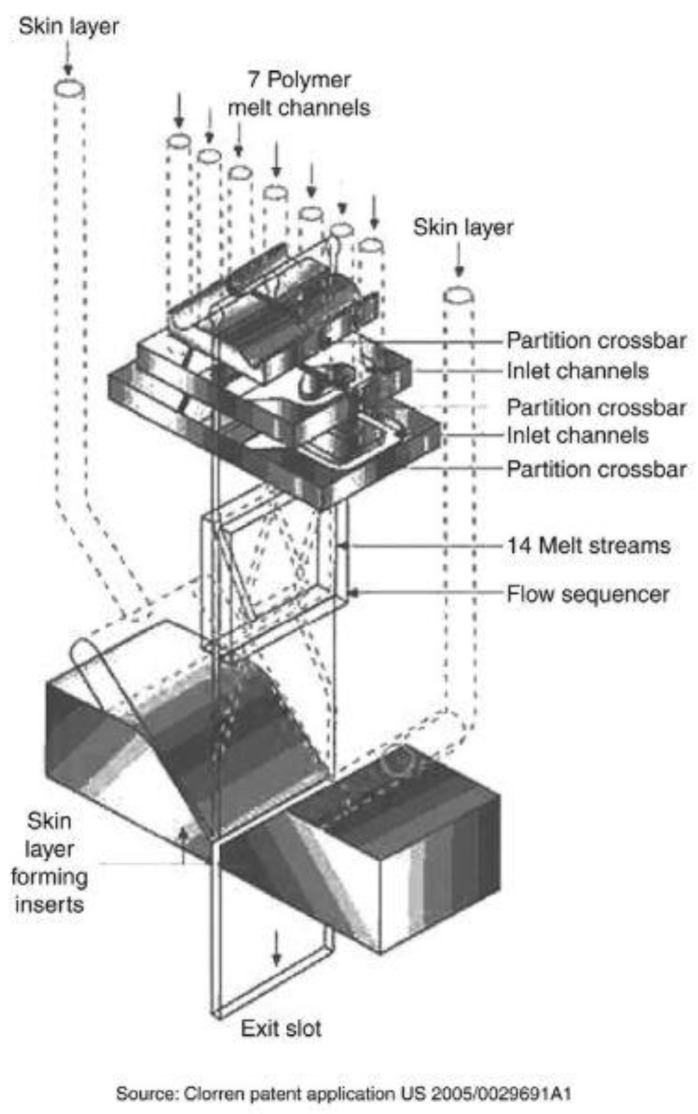
Multi-nano-layers feedblock design by Cloeren Incorporated. Adapted from [84].

The ability to increase the layer number comes from the feedblock design. In 2002, a single feedblock and film die system reached the micro and nanolayer scale. In Figure 10, a Nanolayer™ feedblock designed by Cloeren is displayed. This feedblock was designed to produce directly more than 1000 layers in a single unit. The die connects a selected number of extruders and redistributes the incoming melt streams into hundreds or thousands of layers. These layers are ordered and distributed within the block using a design, which was inspired from vein splitting. Then, the ordered thousand-layer polymer melts flows, and exits the feedblock directly into the die in order to form the product film or sheet [81,84].

Before the fabrication of single shot feedblocks, Dow Chemical Company developed a combination processing technique using a simple two to five layered feedblock with a series of sequential layer multiplication dies. In this approach of sequential layer multiplication, the two to five layered polymer melt flows through a conventional feedblock and then is fed to a series of layer multiplication dies. These layer multiplication dies double the number of layers by a cutting process, spreading and stacking the layered melt stream (Figure 10). As shown in Figure 10, the final number of layers in the polymer film is determined as the function of a number of layer multiplication dies, which are placed in series between the feedblock and final film or sheet exit dies. The number of layers of the film obtained can be calculated as a function of the number of layers in the feedblock and the number of layer multiplying dies [78,81,84].

**Figure 11:**
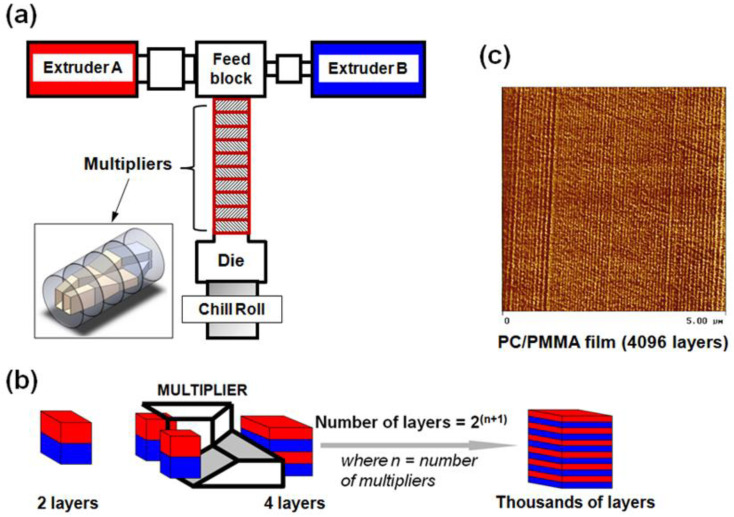
Schematic illustrations of (**a**) multilayer coextrusion process with two polymeric components with a specific scheme of multiplier element used in our laboratory, and (**b**) general layer multiplication concept schematic of the layer multiplication by cutting, spreading and recombining. (**c**) AFM phase image showing the cross-section of a 4096 layers PC/PMMA (50/50) film as an example for coextruded multilayered polymers. Reprinted from publication [67]. Copyright 2020, with the permission from Wiley. (A color version of this figure can be viewed online).

Figure 11 displays an example of nanolayer film coextrusion. The layer multiplying dies is coupled with a two-layered feedblock that will produce films with a number of layers following a 2^n+1^ model. The “n” represents the number of sequential layer multiplying dies, which are placed in series between the feedblock and film exit die [74]. Layer multiplication enables structures with hundreds or thousands of layers to be produced. A layered melt stream from the feedblock is fed through a series of layer multipliers. In each multiplier, the initial melt stream is divided vertically in two, spread horizontally, and then recombined, while keeping the total thickness of the melt constant, thus doubling the number of layers and reducing the thickness of each layer after each multiplier (Figure 10). Therein, multilayer coextrusion is capable of fabricating films having thousands of layers with individual layer thickness down to the nanoscale at low environmental (solvent-free) and budgetary costs [67,78,81].

A coextrusion of two or more polymers is possible, creating different layer configurations. A configuration ABC represents coextrusion of three different polymers as alternating layers. Meanwhile, an ATBTA configuration represents a tie-layer polymer (T) alternating between polymers A and B. The latter structure can also be combined with skin layers which are normally added after the layer multiplication dies [74].

Comparing the coextrusion approaches, the layer multiplier die technique is a more flexible and low-cost technique than the single shot feedblock. Nowadays, the single feedblock processing technique is used more often in production of commercial scale products. Thus, the sequential layer multiplying die has been used for research and as a development tool. Most of the time, commercial products formulations and structures are designed and optimized before their commercialization with lower cost equipment and production costs [78,81,84].

#### 6.1.2. Blown Film Coextrusion

**Figure 12:**
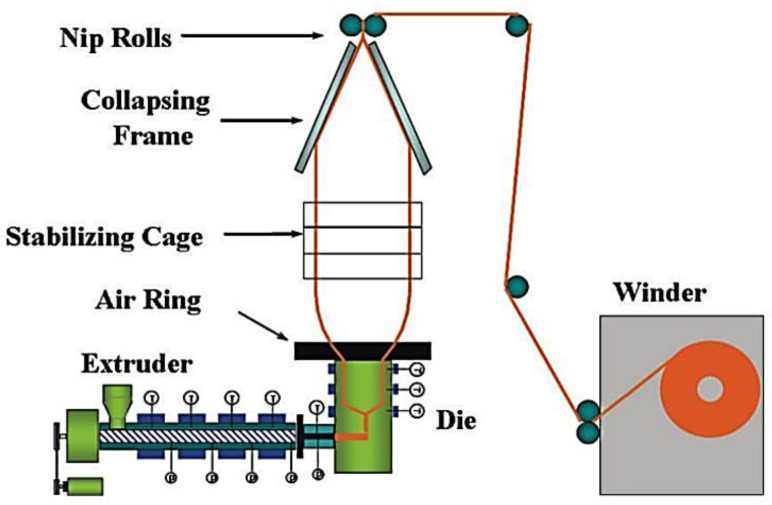
Blown film process schema. Adapted from [85].

Blown film extrusion is widely used to produce packaging films. The majority of these films are multilayered in order to improve its mechanical and thermal properties as required by the medical or food industry. In Figure 12, a schematic diagram of the blown film processes is displayed. As observed in this process, an extruder is used to melt and forward molten polymer into an annular film die. Then, air is injected into the center of the annular die to inflate the polymer bubble. This bubble is cooled down by an air ring, which blows air on the bubble surface to decrease its temperature until the polymer becomes solid. A stabilizing cage is commonly used to minimize the bubble movement as it collapses in the collapsing frame to make a flat film. The film is then pulled over and fed into a film winder to obtain a finished film roll [85].

In recent years, research efforts have been made by Dow Chemical Company and Cryovac/Sealed Air Corporation to develop new technologies in the context of micro and nanolayer coextrusion for blown film processing. The early version of blown film technology used spiral mandrel dies. The layers are made by separate spiral manifolds, which are present at different radial distances [84]. Then, the melt from different manifolds is joined together near the die exit to form a layered structure. In this type of die, to increase the number of layers in the structure, the diameter of the die has to be increased in order to make room for more spiral mandrel manifolds for each new layer. Thus, this tends to limit the number of layers, since larger diameters dies have longer residence times which can lead to the degradation of the polymers being processed [85].

Another style of spiral mandrel die has been developed, where the spiral channels are cut on the surface of a flat plate rather than on the cylinder surface. This design allows multiple overlapping spirals cut into the same plate. The use of stacked plates with spiral channels on surface plates allow stacking multiple plates to create layered structures. Because of the dimensions of these large flat plates, these dies are commonly referred to as “pancake” style dies [85].

**Figure 13:**
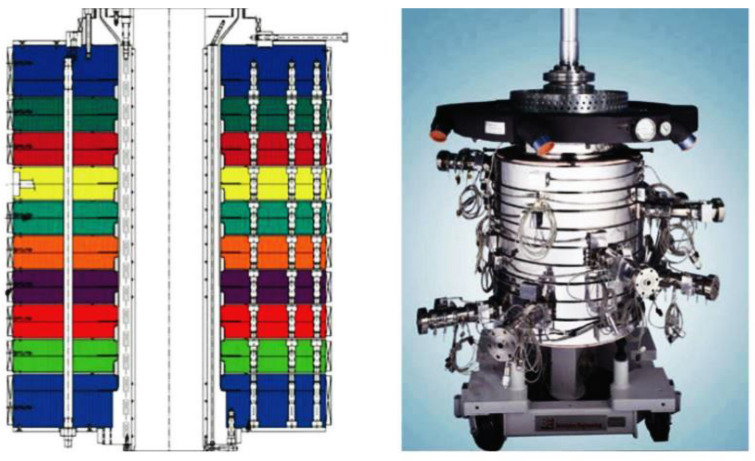
Schematic and commercial example of a multilayer stacked plate or “pancake” die. Adapted from [85].

The advantage of using a flat die for the coextrusion of multilayer films is the ability to stack plates on top of each other. In Figure 13, a schematic diagram of the multiple stacked plates bolted together is shown, where each set of plates produces one layer. The layers are added sequentially to the previous layer as the structure flows up the die towards the exit. An example of a commercial stacked plate die is also displayed in Figure 11, in which it is used to produce coextruded films. Structures containing up to 11 layers have been demonstrated using this type of design. However, increasing the number of layers after 11 layers is challenging, due to the pressure drop and lack of space for more extruders [85]. Multilayered blown film lines are currently commercially available from different equipment manufacturers such as Davis Standard, Macro Engineering, Alpha Marathon, Bandera, Windsor, etc. [84].

The challenge of adapting the feedblock and layer multiplier dies technologies from flat film to annular structures involves ensuring the layers continuity around the circumference of the bubble. No uniform layer thicknesses in the films can be obtained if there are breaks and weld lines during the layer wrapping around the circular dies [84].

Using a feedblock and layer multipliers in combination with a specific film die is the new concept applied to produce microlayers by blown extrusion. There are two important characteristics of the film die to consider:

- Protection of the thinner microlayers as they flow from the feedblock to and around the die.
- Geometry design that allows the layers to flow slowly through the die while maintaining the microlayered structure

**Figure 14:**
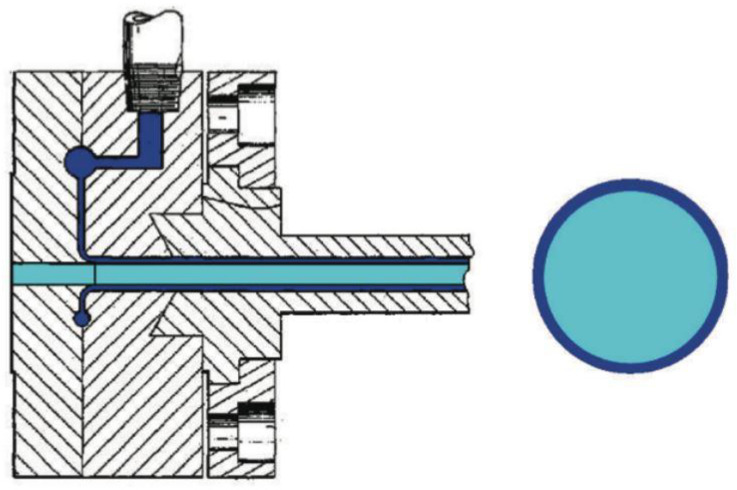
Schema of an encapsulation die (on the left) producing an encapsulated structure (on the right). Adapted from [85].

In order to protect the microlayered structure as it flows through the process, another layer is added to encapsulate the microlayers. In Figure 14, a schema of the encapsulation die is displayed (on the left), which produce a circular encapsulated structure (on the right). This example shows a single core material being encapsulated by another layer. However, in theory, the single core material could be replaced by a microlayer structure [85].

Further research innovations confirmed the processing of 100+ microlayered films in a blown film structure as [85] demonstrated. Developing uniform wrap of sequentially layered films will continue to challenge the layer multiplier die approach for blown film coextrusion [84].

### 6.2. Multilayer Films in Agricultural Applications

In the last decades, the agricultural evolution has been parallel to the technological development associated to the use of multilayer plastic films. For the agricultural market, the use of multilayer plastics films has been essential, allowing a remarkable increase of agricultural productions, earlier harvesting, and reduction of plagues presence [86]. The growing use of plastics in agriculture has been helping the farmers to increase their crop production. Nowadays, the use of plastics allows to increase yields, earlier harvests, less dependence on herbicides and pesticides, better protection of food products, and more efficient water conservation [59]. An extensive expanding of plastic films in agriculture is reported worldwide since the middle of the last century. The most common applications of plastics in agriculture are displayed in Table 5.

Plastic films can improve product quality by mitigating extreme weather changes, optimizing growth conditions, extending the growing season, and reducing plant diseases. An estimated 2–3 million tons of plastics are used each year in agricultural applications. Almost half of the total plastics produced each year for agricultural applications is used in protective cultivation as mulching, greenhouses, small tunnels, temporary covering for fruits trees, etc. [88]. For this application, the most common polymers used are LLDPE followed by LDPE, and EVA [3].

**Figure 15:**
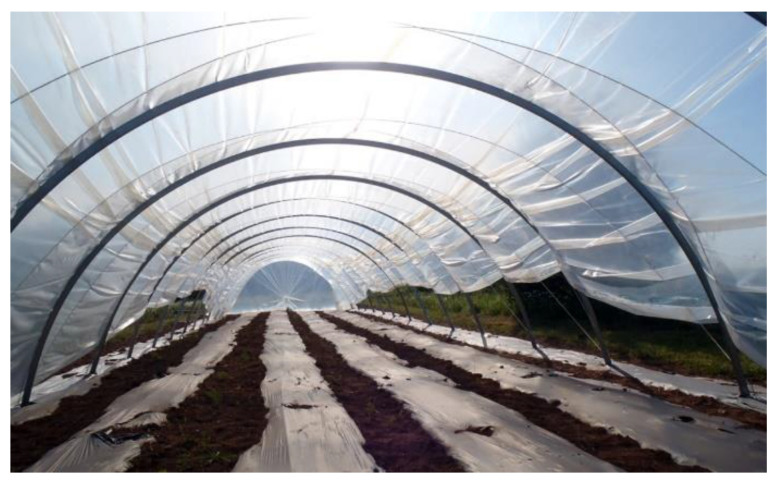
Greenhouse covering. Reprinted from website [89].

**Figure 16:**
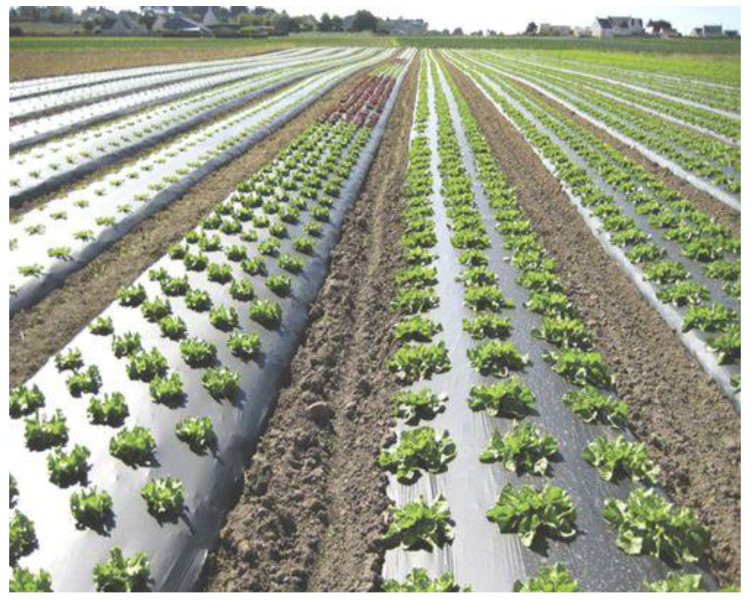
Black Mulch films. Reprinted from website [90].

**Figure 17:**
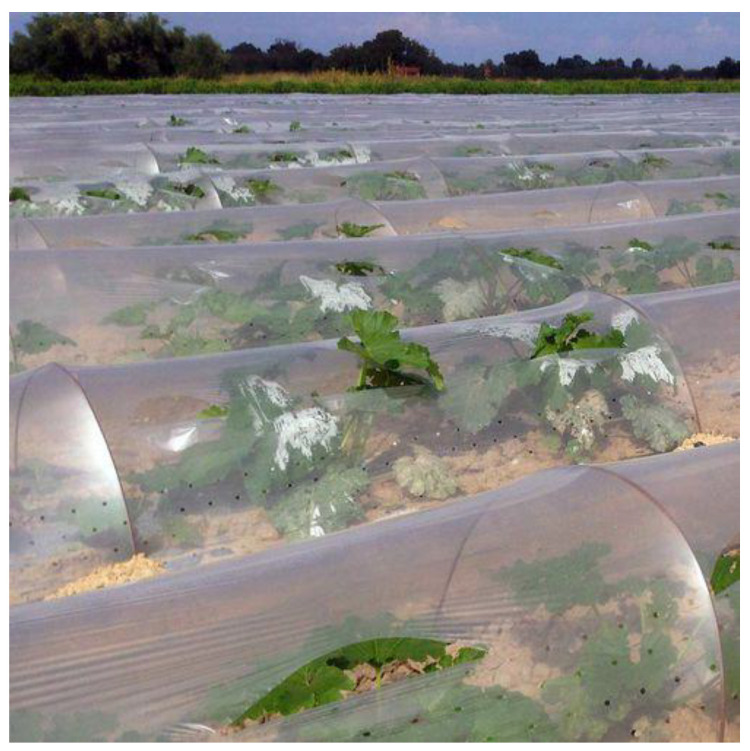
Low tunnel transparent film. Reprinted from website [91].

**Figure 18:**
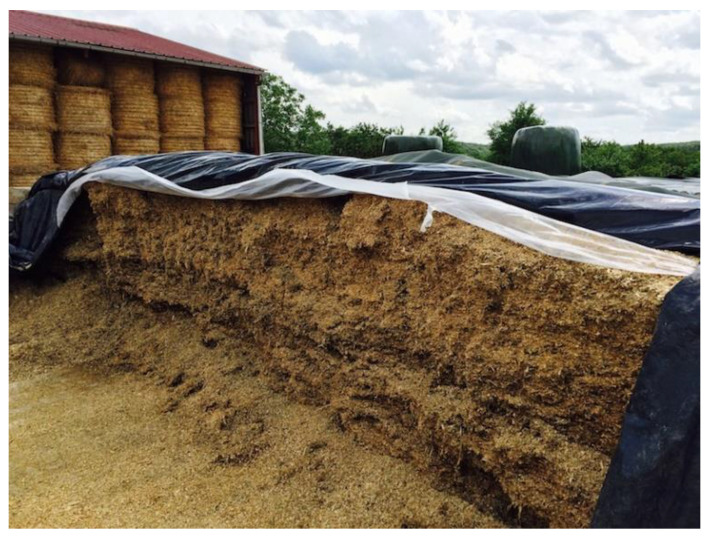
Protection Silage films. Reprinted from website [46].

**Figure 19:**
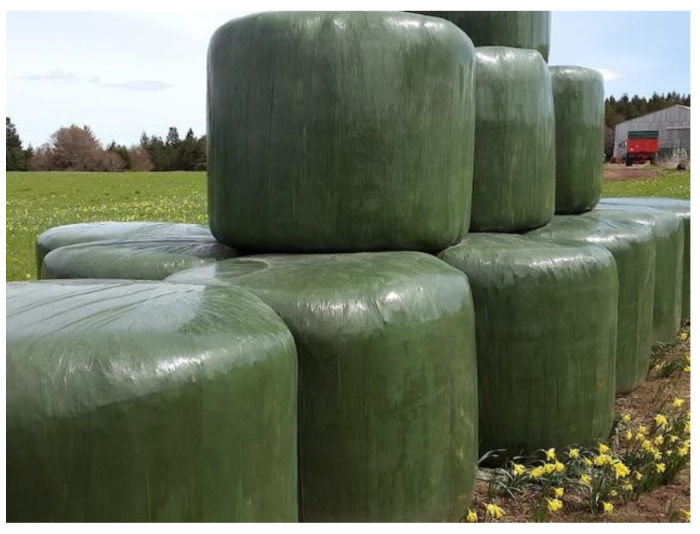
Wrapping stretch films. Reprinted from website [46].

Plastic films used in agriculture are made by blowing coextrusion process. Coextrusion offers many possibilities by combining the properties of different polymers in order to satisfy each agricultural application [86]:

- **Greenhouse covering:** Greenhouse are films used for crop protection (Figure 15). Films with a range of 100 µm to 1 µm that answer the basic requirements of thermal protection, light transmission, and direct or artificial flood lighting.
- **Mulching:** This technique consists in covering the soil (where the cultivation has been planted) with a layer that protects seedlings and young plants (Figure 16). The films used are transparent or opaque, white, colored, or black with range thickness of 20–50 µm, which are mechanically laid on the soil.
- **Low tunnels:** They consist of small arch-shaped support structures covered by plastic films with the objective to create a microclimate suitable for cultivation (Figure 17). Films of about 60–100 µm thickness are generally installed.
- **Silage films:** Silage is a technique used for conservation of wet forage by acidification of the environment protected from the ambient air (Figure 18). Depending on their specific application, films with a range between 35 and 100 µm are installed.
- **Bale wrap films:** Wrapping films are used for individually or continuously wrapping cylindrical or square bales of fodder in order to obtain an airtight envelop, which allow the anaerobic fermentation process necessary for the production of silage (Figure 19). Films can be black, white, and other colors (green and brown) with thickness range of 20–30 µm.

As mentioned before, the most common polymers used in agriculture plastic films are linear low-density polyethylene (LLDPE), linear density polyethylene (LDPE), and Ethylene vinyl acetate copolymer (EVA). The most important properties for the agricultural application of these polymers are shown in Table 6.

Additionally, agriculture films contain additives such as antioxidant, UV-rays, absorbers, fillers, etc. to make them suitable to the application they are aimed for. The main functions of additives are [86]:

- **Plasticizers, lubricants, sliding agents, etc.:** Facilitate the extrusion process.
- **Compatibilizers and coupling agents:** Improve the polymer blends adhesion.
- **Thermal stabilizers and light stabilizers:** Increase the resistance to degradation during the extrusion process or the product life cycle.
- **Cling additives:** Provide adhesive properties to the surface of the films, used mostly for stretch wrap films.
- **Mineral fillers, reinforcements, impact absorbers:** Improve mechanical properties.
- **Pigments, colorants, nucleant agent:** Modify product appearance.
- **Smoke inhibitors, biocides, anti-fog agents, etc.:** Improve the performance of the product.

### 6.3. Polymers Commonly Used in Agricultural Multilayer Films

#### 6.3.1. Linear Low-Density Polyethylene (LLDPE)

Linear low-density polyethylene resin are molecules with linear polyethylene backbone to which short alkyl groups are attached at random intervals. They are produced by the copolymerization of ethylene with 1-alkenes. Ethyl, butyl, or hexyl groups are the most common branches found in these copolymers. However, a variety of other alkyl groups can be also found, both linear and branched. The typical range of average branches separation along the main chain is around 25–100 carbon atoms. Additionally, LLDPE can present low levels of long chain branching (LCB), but not as complex as the ones found in low-density polyethylene [92]. The comonomers used are typically 1-butene, 1-hexene, and 1-octene. The carbon atoms participating in the comonomer vinyl group are incorporated into the polymer backbone. The remaining carbon atoms form pendant groups, which then are referred to as short-branches (SCB).

One of the attractive attributes of LLDPE is its high film tear strength. This property coupled to its relatively high clarity compared to HDPE (high density polyethylene), generate high acceptance as a polymer grade for film packaging and other applications such as agriculture [92].

#### 6.3.2. Low-Density Polyethylene (LDPE)

The LDPEs are polymers, which contain high branching concentration that hinder the crystallization process, resulting in relatively low densities. Ethyl and butyl groups are the branches found in the LDPE backbone together with some long branches. Since these polymers are produced by high pressure polymerization process, the ethyl and butyl branches are frequently clustered together, separated by long unbranched backbones. Long chain branches (LCB) appear at random intervals along the main chain length. The LCB can also turn into branches themselves. Low-density polyethylene polymers have densities typically in the range of 0.90–0.94 g/cm^3^ [92].

Furthermore, the high content of short branches (SCB) found in LDPE reduce its degree of crystallinity, which result in a flexible product with a low melting point. On the other hand, the LCB proportionate desirable processing characteristics, high melt strengths, and relatively low viscosities. These characteristics are attractive for film-blowing processing. The most popular applications of LDPE are low load commercial and retail packaging, and trash bags. Other uses include shrink-wrap, vapor barriers, agricultural ground cover, and greenhouse covers.

#### 6.3.3. Ethylene Vinyl Acetate (EVA)

Ethylene Vinyl Acetate (EVA) is the largest volume of polar ethylene copolymers that are most commercialized. Similar free radical polymerization processes used for LDPE homopolymers are used to obtain EVA polymers. The comonomer vinyl acetate (VA) is distributed randomly along the ethylene backbone and long-chain branches. VA comonomer low crystallinity and high polarity gives the polymer adhesive characteristics and low seal initiation temperature. EVA resins are processed below 230 °C since they are thermally unstable [93].

Since the VA has higher density than the ethylene monomer, it disrupts the polymer crystallinity. This means that the VA increase the polymer density and decrease the crystallinity [93]. The resulting materials have low modulus and good clarity. Additionally, their high branch content results in low lamellar thickness, which translates into low melting temperatures. By contrast, the LCB provide these copolymers with melt characteristics similar to the ones of LDPE. EVA is used generally in packaging films, because of their flexibility, toughness, elasticity, and clarity desirable characteristics. The other main use of ethylene vinyl acetate is as a component for adhesive’s formulation.

#### 6.3.4. Polyisobutylene (PIB)

**Figure 20:**
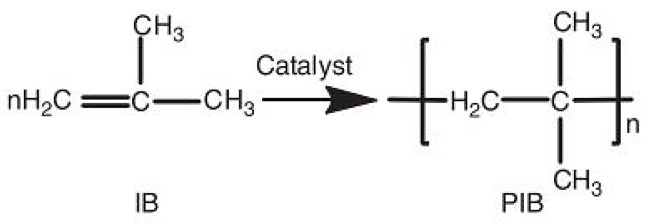
Scheme of the polymerization of isobutylene (IB) to form polyisobutylene (PIB). Adapted from [94].

Polyisobutylene (PIB) is a vinyl polymer produce from a monomer isobutylene (IB) by cationic polymerization (Figure 20). PIB is usually classified as a synthetic rubber or elastomer, despite its linear structure. PIB is a colorless to light yellow, elastic, semisolid, or viscous substance. It has unique properties such as low moisture and gas permeability, good thermal and oxidative stability, chemical resistance, and high tack in adhesive formulations. Additionally, it is odorless, tasteless, and nontoxic. Because of their nonpolar structure, PIBs are soluble in aliphatic and aromatic hydrocarbon solvents. The PIB amorphous characteristics and low glass transition temperature (T_g_ ≈ −62 °C) give its high flexibility and permanent tack [94].

PIB applications include adhesives, agricultural chemicals, sealants, cling film, personal care products and pigments concentrates, etc.

PIB is widely used as a basic substance in the compounding of pressure-sensitive adhesives (PSAs). Low-molecular weight PIBs are soft and liquid-like, which makes them suitable tacktifiers. The two most important parameters of PSAs are tack and holding power. The cohesive strength of PIBs is relatively low; it can be increased with the addition of high-molecular weight PIB or fillers. PSAs formulated with PIBs are aging resistant and are employed to give adhesion to a variety of substrates such as polyethylenes films for cling films applications [94]. In order to obtain the desired PSA properties, different approaches can be obtained. A combination of low molecular weight and high molecular weight PIBs is used to reach a balance between the cohesive and tack strength. Typically, 80 wt% of low molecular weight PIB is combined with high molecular weight PIB, in order to obtained PSAs with fairly mild adhesive characteristics.

PIB based PSAs are widely used in well-establish application areas such as building, packaging, and tapes. Thanks to their nontoxicity, it is approved for food/food contact, and thus the special interest for food packaging and agricultural applications [94].

## 7. Main Conclusions and Prospects

The increasing generation and accumulation of non-biodegradable waste is becoming a high-profile public issue, since huge amounts of plastic packaging products are currently designed to have a short service life owing to their low cost and easy production. Unfortunately, most consumers have a negative perception of plastic packaging because of the considerable amount of waste produced in their daily lives. All things considered, it is of great importance to find new solutions for the valorization of multilayered films.

It is a daunting task to summarize the recent and intensive papers published in the last 3 years regarding the subject. However, to the best of our knowledge, there are few works dedicated to (i) eco-design in order to improve recyclability by mechanical recycling and (ii) the recycling of multilayer films in general. This is a reason why a special attention is given to those approaches to complete the pool of the recent papers in this field. Hence, this review focused on the summary of plastic waste management in general, making emphasis on the multilayer film’s recycling process. Then, the multilayer film’s manufacturing process was described, including the most common materials used for agricultural applications, their processing, and the challenges of their recycling.

Recycling is a key factor to close the loop in the circular economy. However, the fact that multilayer films are made of different types of polymers causes their recycling to be even more challenging. Considering that complex blends are obtained after the mechanical recycling process, compatibilizers need to be added into the blends, but this process can create new issues that need to be further solved. Hence, a novel approach from eco-design to recycling of multilayer structures should be considered as an alternative if mechanical recycling can be assumed.

In addition to chemical recycling, which is one of the promising solutions in the future, the use of strategies such as structure simplification and eco-design are key factors in increasing the recyclability of multilayer plastic films.

## Figures and Tables

**Table 1:** List of definitions of different collection systems [35].

TERMINOLOGY	DEFINITION
**Kerbside Collection**	Separate collection system, categorized as property close collection
**Mixed Solid Waste (MSW)**	Collected in a system in which no other separate collection is present.
**Bring Points**	Common collection points are shared by a larger number of citizens and involve individual transport to deliver the recyclable waste materials

**Table 2:** Plastic flexible films collection schemes and programs in European countries ([3,35]).

Collection Type		Materials	EU Countries	Films Collection
Kerbside	Single fraction	Plastic	Austria, Netherlands	Comingled flexible and rigid plastic collection
	Co-mingled	Plastic and metal	Germany, Slovenia, Hungary	Collected with mixed plastics
			France	Some collections with mixed plastics
			Italy	Rigid and film plastics are collected separately
		Glass, plastic and metal all in one bin	Ireland	Collected with mixed recyclables
Bring points	Single Fraction	Plastic	Sweden	Collected with mixed plastics
	Co-mingled	Plastic and metal	Spain, Portugal	Collected with mixed plastics
Retail return system (drop-off)	-	Plastic	United Kingdom	Plastics PE films collected separately

**Table 3:** Summary of some chemical recycling techniques for plastic waste, incorporating their advantages and challenges [20].

Techniques	Advantages	Challenges
Chemolysis	Generates pure value-added products	To be cost-effective requires high volumes
Pyrolysis	Simple technology and suitable for highly heterogeneous plastics blends	Complexity of reactions
Fluid Catalytic cracking	Economically favorable since the reaction conditions are less strict	Absence of suitable reactor technology
Hydrocracking	Suitable for plastic blends. Good quality of the produced naphta	High operational cost Presence of inorganics High cost of Hydrogen
Gasification	Well-known technology	Generation of noxious NO_x_

**Table 4:** Summary of mechanical recycling and deinking process of post-industrial monolayer waste films [3].

Waste Treatment	Mechanical Recycling System	Type of Extruder System	Description
Closed-loop recycling	Deinking	Cadel Deinking (Spain)	Water-based. High quality recycled pellets obtained for high-added value products
Open-loop recycling	Re-extrusion	Degassing extruders EREMA (Austria)	One stage. Loss of properties. Less demanding applications
	Deinking	Gamma Meccanica (Italy)	Detergents, solvents. Limited to plastic rolls. High costs
		Geo-Tech (US)	Water-based, work with plastic flakes but focused mainly on rigid plastics.
		Metalurgica Rhaaplex (Brazil)	Solvent-based solution and friction system. Work with plastics flakes. High costs and lower quality of recycled material obtained
		CLIPP+(EU)	Carbon dioxide in supercritical conditions. Still in development.

**Table 5:** Most common applications of plastic films in agriculture. Reprinted with the permission from [87]. (Copyright 2012, Pagepress).

Protective Cultivation Films	Livestock Farming Films
Low tunnel Greenhouse and tunnel Mulching Direct covering Nursery film Covering vineyards	Silage films Bale wrapping films Protection films

**Table 6:** Main properties of the polymers used in agricultural plastic films applications. Adapted from [86].

Properties	LDPE	LLDPE	EVA
Photostability	+/−	+/−	+/−
Transparency	−	−	+
Mechanical properties	+/−	+	+
Creep	+	+	−
Welding	+	+	+
Extrusion	+	−	+
(+) good; (+/−) intermediate; (−) bad			

## Data Availability

Not applicable.
